# Hugan Buzure Induces Autophagy and Apoptosis in Hepatocellular Carcinoma by Inhibiting PI3K/Akt/mTOR Signaling Pathway

**DOI:** 10.1155/2022/1618491

**Published:** 2022-12-19

**Authors:** Xiaoyi Meng, Min Li, Ming Qiao, Xue Li, Jianhua Yang, Junping Hu

**Affiliations:** ^1^College of Pharmacy, Xinjiang Medical University, Urumqi 830017, China; ^2^Department of Pharmacy, The First Affiliated Hospital of Xinjiang Medical University, Urumqi 830054, China

## Abstract

This study explored the effects of Hugan Buzure (HBR) on cell apoptosis and autophagy in hepatocellular carcinoma (HCC) and the molecular mechanisms of the PI3K/Akt/mTOR signaling pathway. HepG2 and Huh7 cell viability was detected by the tetramethylazolium salt colorimetric (MTT) method. Cell proliferation was measured using the colony formation method. Hoechst 33258 staining and flow cytometry were employed to detect apoptosis. In addition, immunofluorescence was carried out to evaluate the expression of LC3. Western blot was performed to detect the expression of Bcl-2, Bax, Caspase-3, LC3, Beclin1, p62 (SQSTM1), and PI3K/Akt/mTOR signal pathway-related proteins in HCC cells. This work verified that HBR reduced HepG2 and Huh7 cell proliferation in a concentration-dependent manner. Treatment with HBR caused an obvious improvement of the apoptosis rate, accompanied by the increase in Bax/Bcl2, Caspase3, LC3II, and Beclin1 levels, respectively. Furthermore, HBR downregulated the expression of p62, p-PI3K, p-Akt, and p-mTOR proteins. HBR combined with HCQ enhanced HBR-induced apoptosis. In conclusion, HBR induced autophagy and apoptosis through PI3K/Akt/mTOR signaling pathway, leading to HCC cell death. This research preliminarily suggested the potential role of HBR in the treatment of HCC.

## 1. Introduction

Hepatocellular carcinoma (HCC) is the main type of primary liver cancer and the third leading global cause of death [1]. At present, therapeutic regimens for HCC include liver resection, liver transplantation, and other methods, but the high proliferation, metastasis, and high recurrence rate of HCC lead to low treatment effectivity. The prognosis of HCC is limited by tumor heterogeneity, inadequate understanding of the pathology and mechanism of physiology of HCC, and the absence of effective treatments [2]. Currently, synthetic chemical medications (e.g., sorafenib and oxaliplatin) can increase the overall survival rate of HCC patients. However, these medications also have some drawbacks, including diarrhea, hand-foot skin reaction, rash, nausea, and drug resistance. Therefore, the search for innovative, effective, and low-toxicity drugs is urgently needed.

Traditional Chinese medicine (TCM) has been widely used by a large number of cancer patients as complementary and alternative medicine because of its efficacy and lack of serious toxic side effects [3]. For example, Huaier granule reduced the postoperative rate of recurrence of HCC and prolonged the survival rate of patients [4]. *Antrodia cinnamomea* inhibited the expression level of PI3K/Akt and downregulated the production of proteins associated with the cell cycle, helping to slow the progression of HCC [5]. Numerous studies confirmed that TCM has a well-recognized impact on both the prevention and treatment of HCC. TCM is a prospective approach for encouraging apoptosis, autophagy, necrosis, cycle disruption, growth inhibition, proliferation, and invasion of cancer cells rather than directly killing cancer cells.

The traditional Uygur medicine Hugan Buzure (HBR), a classic prescription in the clinical treatment of liver diseases in Xinjiang, China, has a long historical standing and good clinical effect. HBR is clinically used to treat acute and chronic hepatitis B, acute and chronic cholecystitis, and cholangitis. Recent studies have shown that HBR has a good protective effect in CCl_4_-induced liver fibrosis rats and acute liver injury in wine-deprived mice [6, 7]. HBR consists of Cichorii Radix Et Semen, Cuscutae Semen, Foeniculi Fructus, Apii Radix Et Semen, and Foeniculi Cortex. The 75% ethanol extract of Foeniculi Fructus effectively triggers HCC cell apoptosis and inhibits the development of xenografted HCC [8]. Total flavonoids of *Cuscuta chinensis* Lam. may have an inhibitory effect on HCC cell growth depending on the upregulation of the expression of the tumor suppressor gene PTEN in HCC cells (HepG2) by cutting off proliferation, invasion, and other changes in tumor cells generated by Akt activation [9]. Another study reports that apigenin, the principal component of Apii Radix Et Semen, had an anti-hepatocarcinoma effect by inhibiting the proliferation of HCC, inducing apoptosis, and increasing sensitivity to chemotherapy drugs [10]. Daucosterol, the main component of the Foeniculi Cortex, enhances the inhibitory effect on the activity of HepG2 cells by affecting the Wnt/*β*-catenin pathway and regulating the expression of apoptosis-related proteins [11]. The active components, targets, and synergistic mechanisms of HBR in the treatment of liver fibrosis have been proven in our previous investigations [12]. Apigenin reverses liver fibrosis by activating the PI3K/Akt pathway, suppressing MAPKs, and consequently reducing the expression of HIF-1*α* [13]. These results suggest that the main components of HBR have shown anti-HCC activity. However, the predominant role of HBR in anti-HCC is not fully understood.

Autophagy and apoptosis are important for the occurrence and development of HCC. To effectively treat HCC, detecting autophagy, apoptosis, and the regulatory factors and signal transmission pathways involved in HCC is crucial. As the aspartate proteolytic enzyme-3 (caspase-3)-dependent regulation of cell death, apoptosis plays an important role in growth, development, and balance in most cells [14]. Apoptosis by activation is one of the key features of antitumor drugs. By contrast, autophagy is the intracellular degradation system in eukaryotic cells [15]. During the autophagy process, intracellular pathogenic bacteria and damaged organelles are encased in double membranes and transported to lysosomes for destruction. Autophagy dysfunction is associated with several diseases, including cancer, neurodegenerative diseases, and diabetes [16]. Autophagy regulation can trigger multiple cascade reactions that can be applied to tumor treatment. Autophagy and apoptosis regulate tumor cell proliferation and survival. Depending on the cellular environment, autophagy may lead to apoptosis or survival. Autophagy can occur before or concurrently with apoptosis [17]. The interaction of the two processes promotes programmed cell death and maintains body stability. In addition, PI3K/Akt/mTOR, one of the classic tumors signaling pathways, has been proven to play an antitumor role by regulating apoptosis and autophagy [18].

In summary, this work intends to offer a theoretical foundation for the clinical treatment and application of HBR by investigating (a) the effect of HBR on HCC in vitro and (b) the potential molecular mechanism of HBR against HCC.

## 2. Materials and Methods

### 2.1. Cell Culture

The Huh7 cell line was obtained from the Procell Life Science and Technology Company (Wuhan, China). The HepG2 cell line was donated by the laboratory of pharmaceutical chemistry of Xinjiang Medical University. The HepG2 and Huh7 cells were cultured in DMEM medium supplemented with 10% FBS and 1% penicillin/streptomycin (Biological Industries, Israel). Two cells were grown at 37°C in a humidified atmosphere containing 5% CO_2_ (Thermo, USA).

### 2.2. Chemicals and Reagents

Cichorii Radix Et Semen (JJZ-YP-200527, JJZ-YP-191209), Foeniculi Fructus (XHX-YP-170803), and Foeniculi Cortex (HXGP-YP-210115) were purchased from Xinjiang Baokang Uygur Pharmaceutical Co. Ltd. Apii Radix Et Semen (Z30142203, G30131002) was ordered from Xinjiang Uygur Hospital. Cuscutae Semen (150701) was obtained from Xinjiang Heji Chinese Herbal Pieces Co. Ltd. All herbs were authenticated by the pharmacist in the Pharmacy Department of the Xinjiang Medical University. Oxaliplatin (OXA, 100584–202005) was purchased from the National Institutes of Food and Drug Control (Beijing, China). Anti-LC3 (14600-1-AP), anti-p62 (66184-1-lg), anti-Caspase3 (66470-2-lg), anti-bax (60267-1-lg), anti-Beclin1 (66665-1-lg), anti-p-Akt (28731-1-AP), anti-*β*-actin (66009-1-lg), and anti-rabbit and anti-mouse antibodies were obtained from Proteintech (Wuhan, China). Anti-Bcl2 (32124) and mounting medium with DAPI (ab104139) were purchased from Abcam (Cambridge, UK). Anti- PI3K (bs-10657R) and anti-Akt (bs-52010R) were ordered from Bioss (Beijing, China). Anti-p-PI3K (AP0854) was obtained from ABclonal (Wuhan, China). Anti-mTOR (AF6308) and anti-p-mTOR (AF3309) were purchased from Affinity Biosciences (USA). Chloroquine (HCQ) was ordered from MedChemExpress (USA).

### 2.3. Preparation of HBR Aqueous Extract

For the extract, 26 g of Apii Radix, 26 g of Cichorii Semen, 26 g of Foeniculi Cortex, 13 g of Cichorii Radix, 13 g of Foeniculi Fructus, 13 g of Apii Semen, and 6.5 g of Cuscutae Semen were pulverized and mixed with water decoction 3 times for 2 h, 1.5 h, and 1 h. The dry extract was obtained by filtering, concentrating, and drying the filtrate. The yield was 21.7%. The appropriate amount of dry HBR extract was weighed, dissolved in the complete medium, and diluted to obtain the drug concentration for the experiment.

### 2.4. Cell Viability Assay

HepG2 and Huh7 cells (3 × 10^3^ cells per well) were incubated in 96-well plates. The cells were treated with 200 *μ*L of HBR (0.5, 1.5, 2.5, 3.5, 4.5, and 5.5 mg/mL) and OXA (20 *μ*g/mL) for 24, 48, and 72 h. After incubation, 10 *μ*L of MTT (5 mg/mL) solution was added to each well. The plates were then incubated at 37°C for 4 h. Subsequently, 150 *μ*L of DMSO reagent was added to each well to dissolve formazan. The OD was measured at 490 nm using a microplate reader (Thermo, USA).

### 2.5. Colony Formation Assay

HepG2 and Huh7 cells (200 cells per well) were seeded in 12-well plates, cultivated overnight, and then incubated for 48 h with HBR (0, 0.5, 2.5, and 4.5 mg/mL) and OXA (20 *μ*g/mL). Subsequently, the cells were placed in a drug-free medium for 7–10 days. The plates were fixed for 10 min with 4% paraformaldehyde and then incubated for 15 min with 0.1% crystal violet. The cells were washed with PBS for 15 min. Colonies were then counted using ImageJ software.

### 2.6. Hoechst 33258 Staining

HepG2 and Huh7 cells were seeded in 48-well plates per well. Cells were then cultivated overnight and incubated for 48 hours with HBR. After being rinsed with PBS, plates were fixed for 10 minutes with 4% paraformaldehyde and then incubated for 20 minutes with Hoechst 33258 staining solution (Beijing, China). The cells were then observed under a fluorescence microscope (Leica, Germany) and photographed.

### 2.7. Mitochondrial Membrane Potential Assay

HepG2 and Huh7 cells were seeded in 48-well plates, and then HBR was added for 48 h. After being rinsed with PBS, the supernatant was discarded and the plates were stained with the JC-1 dyeing working solution for 20 min (Beijing, China). The cells were then washed with JC-1 staining buffer. Subsequently, PBS was added to the observed cells under a fluorescence microscope and photographed.

### 2.8. Apoptosis Analysis

HepG2 and Huh7 cells were seeded in 48-well plates. The cells were then cultivated overnight and then incubated for 48 h with HBR. After treatment, the cells were collected washed twice in cold PBS and resuspended in a labeling solution. Next, 5 *μ*L of Annexin V and 5 *μ*L of PI were added to the buffer and mixed in the dark at room temperature (New Jersey, USA). The percentage of the apoptosis rate of cells was determined by flow cytometry (BD, USA).

### 2.9. Immunofluorescence Assay

HepG2 and Huh7 cells were seeded in 48-well plates. The cells were then cultivated overnight and then incubated for 48 h with HBR. After three washes, the cells were fixed for 10 min with 4% paraformaldehyde, followed by 0.5% triton X-100 incubation. After blocking nonspecific sites with 1% goat serum, the cells were incubated with the primary antibody LC3 (1 : 200) overnight. After incubation with GoraLite488 conjugated goat anti-rabbit IgG antibody (1 : 200) for 1 h at room temperature, the nuclei were counterstained with DAPI. Then, the image of the cell was observed with an inverted fluorescence microscope.

### 2.10. Western Blot Assay

The HepG2 and Huh7 cell protein was isolated in protein lysate buffer (Solarbio, China), and the BCA protein assay kit was used for protein measurement (Solarbio, China). Protein samples were divided, run through SDS-PAGE, and transferred to PVDF. The membranes were blocked for 2 h in 5% skim milk, incubated with LC3 (1 : 3000), p62 (1 : 5000), Bax (1 : 10000), Bcl-2 (1 : 3000), Caspase3 (1 : 2000), Beclin1 (1 : 10000), p-Akt (1 : 5000), Akt (1 : 5000), mTOR (1 : 500), p-mTOR (1 : 500), p- PI3K (1 : 1000), PI3K (1 : 1000), and *β*-actin (1 : 10000) overnight at 4°C. The protein bands were then probed with HRP secondary antibodies (1 : 5000) for 1 h at room temperature. Finally, an ECL detection device was used to capture these protein bands (Proteinsimple, USA). ImageJ was employed to assess the band densities and adjust protein expression levels on the basis of actin expression.

### 2.11. Statistical Analysis

All data in the study were depicted in the form of mean ± SD for each experiment. Data analysis was analyzed using SPSS 22.0 software. Statistical differences of multiple group comparisons were performed with one-way ANOVA. A *p* value of less than 0.05 was considered to indicate a statistically significant difference.

## 3. Results

### 3.1. HBR Suppressed the Viability and Proliferation of HCC Cells

**Figure 1:**
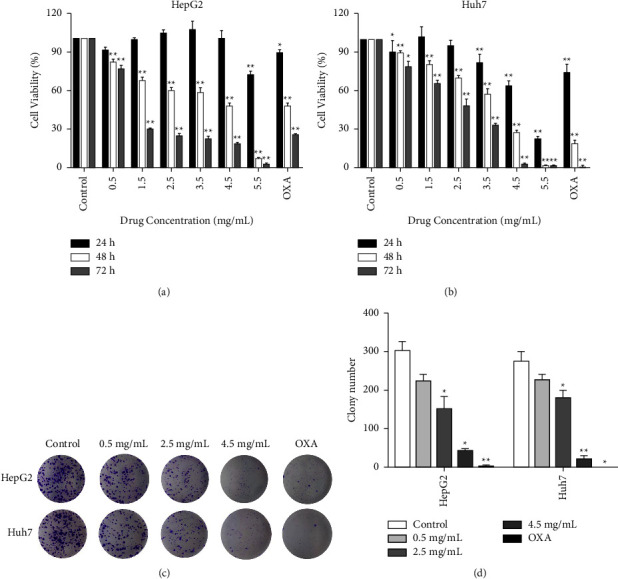
HBR inhibited growth and proliferation in HCC cells. (a) The MTT assay was used to measure the cell viability of the HepG2 cell line treated with HBR. (b) The MTT assay was used to measure the cell viability of the Huh7 cell line treated with HBR. (c) HepG2 and Huh7 cells were exposed to HBR to assess cell colony formation. (d) Quantitative data for (c). Values were shown as mean ± SD in three independent experiments. ^*∗*^*P* < 0.05 and ^*∗∗*^*P* < 0.01 versus the control group.

The antitumor effects of HBR-treated HCC cells were detected by the MTT assay. Between the two cell lines, HBR had a significant concentration-dependent inhibitory effect at 48 and 72 h after administration (Figures 1(a) and 1(b)). However, HBR treatment induced relatively much lower toxicity in LO_2_cells than in HCC cells (Supplementary Figure 1). We chose doses of 0.5, 2.5, and 4.5 mg/mL, and the action time was 48 h for subsequent experiments. Colony formation assays confirmed that HBR had reasonable inhibition effects on HCC cell growth (Figures 1(c) and 1(d)). The data indicated that HBR suppressed cell viability and proliferation in HCC cells but not in normal human hepatocyte cell line LO_2_ cells.

### 3.2. HBR Promoted Apoptosis in HCC Cells

**Figure 2:**
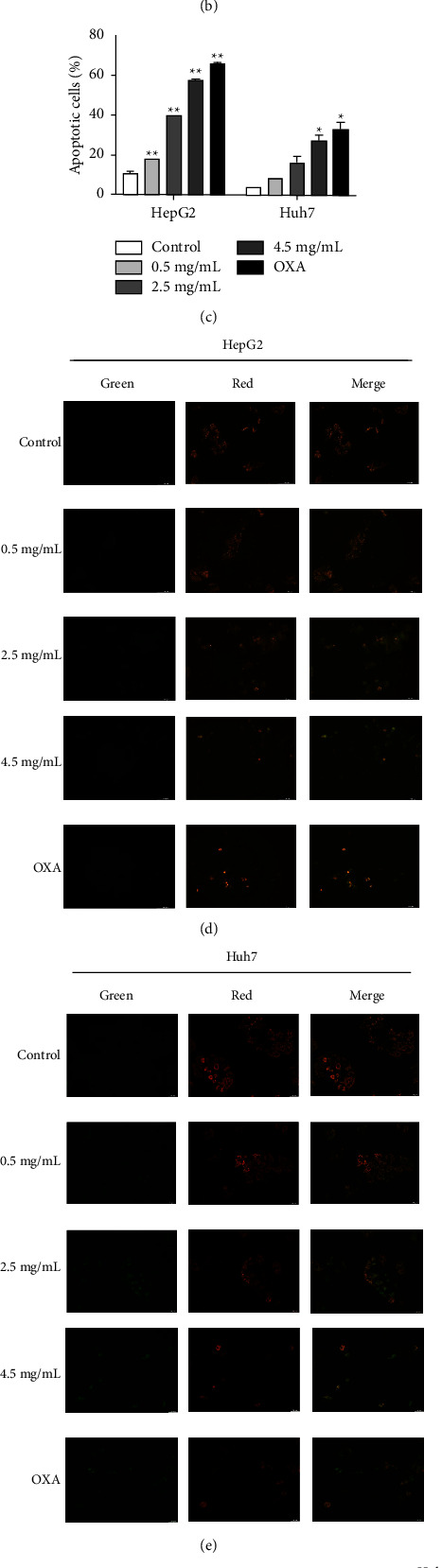
HBR induced apoptosis in HCC cells. (a) HepG2 and Huh7 cells were incubated with various concentrations of HBR. Apoptosis was evaluated by Hoechst 33258 staining. Original magnification: ×200. (b) Apoptosis was analyzed by flow cytometry after double staining with Annexin V/PI. (c) Quantitative data for (b). (d) Effects of HBR on the mitochondrial membrane potential in HepG2 cells. Original magnification: ×200. (e) Effects of HBR on mitochondrial membrane potential in Huh7 cells. Original magnification: ×200. (f) Effects of HBR on the protein levels of Bax, Bcl2, and Caspase3 in HepG2 cell. (g) Effects of HBR on the protein levels of Bax, Bcl2, and Caspase3 in Huh7 cell. (h) Quantitative data for (f). (i) Quantitative data for (g). Values were shown as mean ± SD in three independent experiments. ^*∗*^*P* < 0.05 and ^*∗∗*^*P* < 0.01 versus the control group.

To investigate whether HBR decreased cell viability and proliferation by triggering apoptosis, we treated HepG2 and Huh7 cells with HBR. We performed Hoechst 33258 staining to observe the morphological changes of apoptosis. Compared to the control group, each administration group appeared densely stained with bright blue fluorescence, and the effect was dose-dependent (Figure 2(a)). HCC cells were stained with Annexin V/PI to analyze apoptosis. Results indicate that HBR could increase apoptosis in a concentration-dependent manner (Figures 2(b) and 2(c)). Furthermore, after administration, the mitochondrial membrane potential of HCC cells decreased (Figures 2(d) and 2(e)). Note that an early landmark event of apoptosis is a reduction in the potential of the mitochondrial membrane. Western blot analysis revealed that HBR treatment increased the expression level of Bax/Bcl2 and Caspase3 in HCC cells in a dose-dependent manner (Figures 2(f)–2(i)). These outcomes confirmed that HBR promoted HCC cell apoptosis.

### 3.3. HBR Triggers Autophagy in HCC Cells

**Figure 3:**
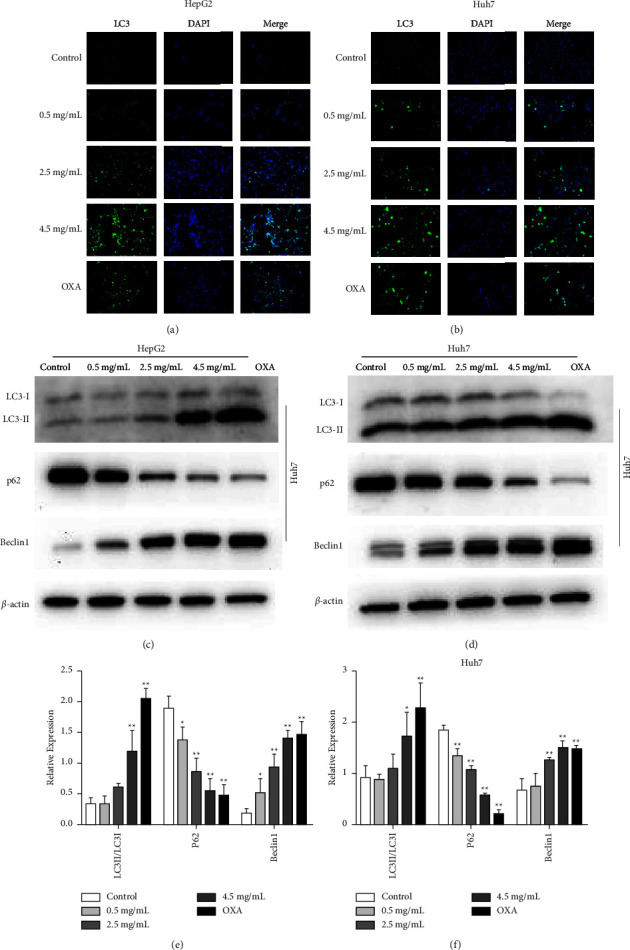
HBR triggered autophagy in HCC cells. (a) HepG2 cells were incubated with various concentrations of HBR. The level of LC3 in cell lines was analyzed with an inverted fluorescence microscope (blue, DAPI; green, LC3). Original magnification: ×200. (b) Huh7 cells were incubated with various concentrations of HBR. The level of LC3 in cell lines was analyzed with an inverted fluorescence microscope (blue, DAPI; green, LC3). Original magnification: ×200. (c) Effects of HBR on the protein level of LC3, p62, and Beclin1 in HepG2 cells. (d) Effects of HBR on the protein level of LC3, p62, and Beclin1 in Huh7 cells. (e) Quantitative data for (c). (f) Quantitative data for (d). Values were shown as mean ± SD in three independent experiments. ^*∗*^*P* < 0.05 and ^*∗∗*^*P* < 0.01 versus the control group.

We identified LC3-II conversion to investigate the impact of HBR on autophagy in HepG2 and Huh7 cells. Using immunofluorescence, we evaluated the intracellular distribution of LC3 puncta. Compared to the control group, HBR improved LC3 positive puncta in HCC cells. Thus, HBR increased intracellular autophagosomes (Figures 3(a) and 3(b)). We evaluated the processing of the autophagosome marker LC3 in HBR-treated HepG2 and Huh7 cells, and HBR improved the protein levels of LC3-II. Furthermore, HBR administration dramatically increased the expression of the Beclin1 protein and downregulated p62 activity in HCC cells (Figures 3(c)–3(f)). Taken together, HBR treatment increased autophagic induction in HepG2 and Huh7 cells.

### 3.4. Inhibition of Autophagy Enhances Apoptosis Induced by HBR in HCC Cells

**Figure 4:**
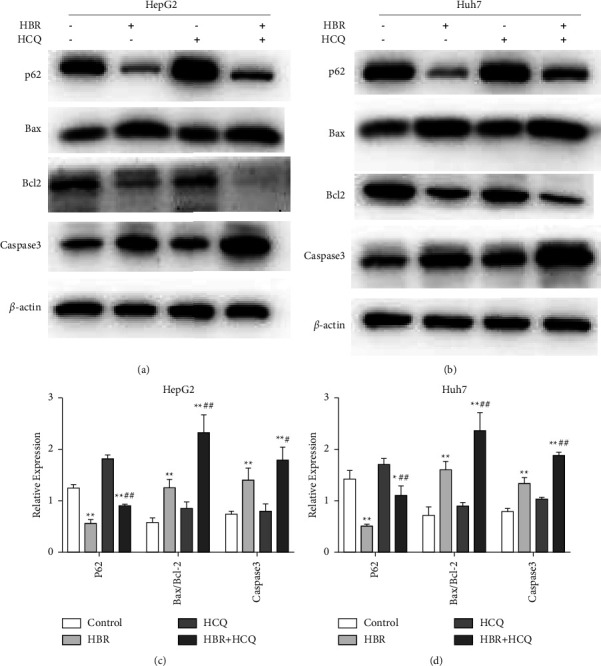
Inhibition of autophagy enhances apoptosis induced by HBR in HCC cells. (a) Effects of HBR treatment with or without HCQ on autophagy and expression of apoptosis-related proteins in HepG2 cells. (b) Effects of HBR treatment with or without HCQ on autophagy and expression of apoptosis-related proteins in Huh7 cells. (c) Quantitative data for (a). (d) Quantitative data for (b). Values were shown as mean ± SD in three independent experiments. ^*∗*^*P* < 0.05 and ^*∗∗*^*P* < 0.01 versus the control group.

To determine the effect of HBR combined with the autophagy inhibitor HCQ on apoptosis of HCC cells, the autophagy inhibitor HCQ was employed to investigate the cells. HCQ reduced the inhibitory influence of HBR treatment on p62 expression. The combination of HBR and HCQ clearly increased the expression of Bax/Bcl2 and Caspase3 (Figures 4(a)–4(d)). The above results established that when the autophagy induced by HBR was blocked, HBR enhanced the apoptosis of HCC cells.

### 3.5. HBR Inhibits the PI3K/Akt/mTOR Signaling Pathway

**Figure 5:**
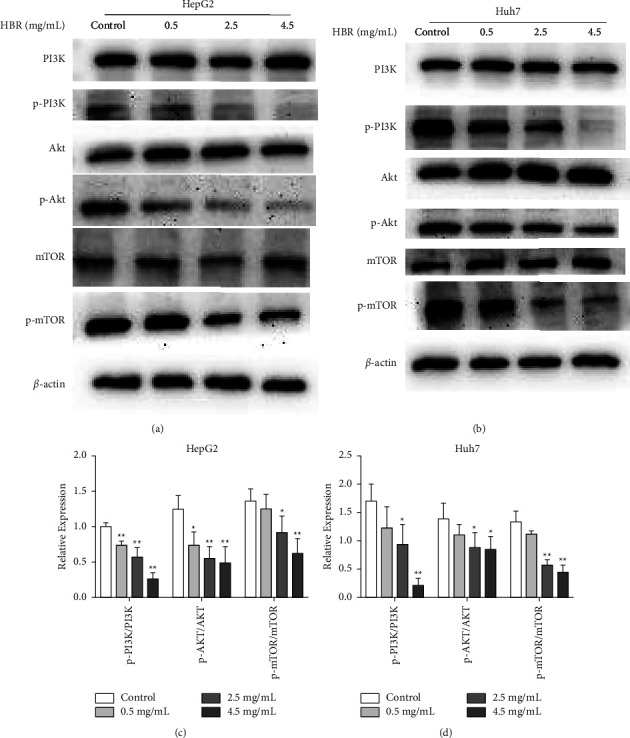
HBR inhibits the PI3K/Akt/mTOR signaling pathway. (a) Effects of HBR on the PI3K/Akt/mTOR-related protein expression in HepG2 cells. (b) Effects of HBR on the PI3K/Akt/mTOR-related protein expression in Huh7cells. (c) Quantitative data for (a). (d) Quantitative data for (b). Values were shown as mean ± SD in three independent experiments. ^*∗*^*P* < 0.05 and ^*∗∗*^*P* < 0.01 versus the control group.

The PI3K/Akt/mTOR signaling pathway plays a pivotal role in the regulation of autophagy and apoptosis. Western blot was performed to detect the expression of PI3K/Akt/mTOR signal pathway-related proteins in the HCC cell. As shown by the analysis, HBR treatment inhibited phosphorylated Akt, PI3K, and mTOR in a dose-dependent manner (Figures 5(a)–5(d)). Therefore, HBR can regulate apoptosis and autophagy by inhibiting the activation of the PI3K/Akt/mTOR signaling pathway.

## 4. Discussion

HCC is a leading cause of cancer-related death worldwide, and the prevention and treatment of HCC presents a considerable clinical challenge. TCM has strong anticancer activity in cancer prevention and treatment. Patients with liver cancer who use Huaier granules experience a protective effect in terms of survival, and survival time is positively correlated with medication time [19]. The treatment with Fuzheng Huayu Jiedu can effectively improve clinical efficacy in advanced liver cancer with fewer adverse reactions and high safety [20].

This study proved that HBR treatment inhibited the proliferation activity of HCC cells but had low cytotoxicity against normal human hepatocyte LO_2_ cells, thereby indicating that HBR had a selective antitumor action to some degree. Different HCC cells are derived from different tissues of patients with liver cancer, which entail distinct genomes, gene expression profiles, protein expression profiles, and immunogenicity. Investigating different HCC cells can provide a general rather than a relatively one-sided result. Apoptosis is mainly regulated by exogenous apoptotic pathways guided by cell surface death receptors and intrinsic apoptotic pathways mediated by mitochondria. Intrinsic apoptotic pathways are primarily regulated by B-cell lymphoma family 2 [21]. The exogenous apoptosis pathway is mediated by cell surface death receptors. Both pathways activate the caspase cascade, which eventually causes Caspase3 and other important proteins to cleave [22]. Our results showed that HBR exerted cytotoxicity by inducing apoptosis of HCC cells and triggered Caspase3 activation. Consequently, HBR could cause HCC cells to trigger caspase-dependent apoptosis. In response to cellular stress and hypoxia, the activation of several mitochondrial molecular targets including Bcl-2, Bax, and caspases can activate the intrinsic apoptotic pathway. A sharp decline occurred in mitochondrial membrane potential because of the transition to a reducing expression level of Bcl-2 and Bax (Bcl-2/Bax), thus activating cytosolic caspases [23]. Recognition of homologous ligands by specific cell surface receptors, such as the tumor necrosis factor receptor and the Fas ligand receptor, initiates the extrinsic apoptotic cascade. This pathway is activated by signals originating in the extracellular environment [24]. In this investigation, a decrease in the mitochondrial membrane and a reduction in the ratio of Bcl-2/Bax were induced by HBR. Therefore, HBR-induced apoptosis may be regulated by the mitochondria-mediated endogenous apoptotic pathway.

Autophagy, the degradation of superfluous proteins and organelles by lysosomes, is an evolutionarily conserved pathway. Numerous studies have indicated that targeted autophagy can be induced in response to anticancer treatments, supporting the idea that although autophagy is widely recognized to influence cell survival, it can also induce cell death in the course of treating cancer, including HCC [25]. Modulation of autophagy-mediated cell death is one of the mechanisms by which several herbal medications are known to have anti-HCC effects. The Fuzheng Yiliu decoction can suppress liver metastasis in mice tumor growth; induce apoptosis of tumor cells; and activate Beclin1, Bnip3, and LC3 proteins of HCC cells (HepG2s), which induce the autophagy of tumor cells [26]. Hydroxysafflor yellow A has shown inhibitory cell activity and promoted cell apoptosis by preventing autophagic flow in HCC cells and is extracted from the herb *Cathartics tinctorius* L. [27]. Autophagy is in close correlation with the occurrence and development of cancer, and the relationship between the two is multisided [28]. Herein, we demonstrate that HBR induced autophagy in HCC cells. LC3 is currently recognized as a marker of autophagy, and the increased transformation from LC3-1 to LC3-II indicates the activation of autophagy. In this study, the level of the autophagy marker LC3-II/LC3-I was enhanced after HBR administration, and immunofluorescence results also revealed that the LC3 spots were enhanced, indicating that autophagy was activated. P62 is an autophagy marker protein that can exert an autophagy effect in tumor cells. The increase in autophagy flow leads to a decrease in the proportion of p62. This work confirmed that p62 expression decreased after HBR administration, suggesting increased autophagy. The autophagy-related protein Beclin1 plays a crucial role in autophagy and is responsible for activating and initiating autophagy [29]. The increase in the Beclin1 level and the transition from LC3-I to LC3-II are associated with the development of autophagosomes [30]. The findings demonstrated that HBR treatment improved Beclin1 expression. The above results confirm that HBR might trigger autophagy in HCC cells.

Apoptosis and autophagy are the main ways of programmed cell death. Previous studies have confirmed that apoptosis and autophagy are not independent, and some regulatory factors, such as Bcl-2 family proteins, caspase family proteins, and ATG proteins, appear simultaneously in the regulatory network of both sides, suggesting that an interaction between them may exist, including promotion, antagonism, and cooperation [31, 32]. This experiment confirmed that HBR combined with the HCQ autophagy inhibitor increased the rate of apoptosis of cells and increased the expression of Bax/Bcl-2 and Caspase3. Thus, HBR-induced autophagy and apoptosis may be mutually cooperative. Autophagy and apoptosis work together to cause cell death, either simultaneously or separately, i.e., when one of them is inhibited, the other activates and continues to complete the process.

The PI3K/Akt/mTOR pathway is one of the crucial signaling pathways triggered by multiple receptor-tyrosine kinases. Inositol phospholipids can be phosphorylated by PI3K, a member of the lipid kinase family [33]. The Akt serine-threonine kinase is normally found in the cytoplasm; when PI3K is activated, Akt moves to the cell membrane, changing its conformation. In addition to acting in the PI3K/Akt pathway, the highly conserved protein kinase known as mTOR is also essential for controlling tumor cell motility and cancer spread [34]. The PI3K/Akt/mTOR pathway plays a vital role in the development of HCC. Its activation is associated with cell proliferation, autophagy, and apoptosis. The Zhenwu decoction inhibited the rapid growth of the tumor by regulating crucial PI3K/Akt/mTOR molecule and prolonged survival time by improving adrenal and spleen function [35]. The flavonoid isoliquiritigenin (ISL), derived from *Glycyrrhiza glabra*, can regulate both apoptosis and autophagy in HCC, resulting in cell death. Isoliquiritigenin is also related to PI3K/Akt/mTOR signaling [36]. Drugs that target PI3K/Akt/mTOR signaling could affect HCC by regulating the families of apoptosis proteins. With the decrease in the activity of phosphorylated PI3K, Akt, and mTOR, the PI3K/Akt/mTOR pathway in HepG2 and Huh7 cells treated with HBR was inhibited, suggesting that HBR could induce apoptosis and autophagy and that suppression of the PI3K/Akt/mTOR signaling pathway is involved in these effects.

Results of the present study achieved on two HCC cell lines-based in vitro systems confirmed the role of apoptosis and autophagy in mediating the inhibition of HCC cell activity and proliferation by HBR and clarified the further underlying molecular mechanisms. Although the present study provides evidence that HBR has a therapeutic potential to HCC cells in vitro, further studies are still needed to validate the possibility of its application in HCC. For example, it is necessary to validate the therapeutic efficacy of HBR in xenograft animals to develop HBR as a complementary candidate against HCC in humans.

In summary, the results of this study indicated that HBR can inhibit the proliferation of HCC via the regulation of apoptosis and autophagy by modulating the PI3K/Akt/mTOR signaling pathway. In addition, HBR combined with HCQ enhanced HBR-induced apoptosis. All these findings provide insight into the action of HBR and suggest a possible role for it in HCC treatment.

## Data Availability

The data used to support the findings of this study are available from the corresponding author upon request.

## Supplementary Materials

Supplementary Materials Supplementary Figure 1: the cell viability of the LO_2_ cell line treated with HBR.
