# Supplementary material for: Hugan Buzure Induces Autophagy and Apoptosis in Hepatocellular Carcinoma by Inhibiting PI3K/Akt/mTOR Signaling Pathway
Source: Evid Based Complement Alternat Med. 2022 Dec 19;2022:1618491. doi: 10.1155/2022/1618491 (PMC10718767; doi:10.1155/2022/1618491)
Supplement: Supplementary Materials — Supplementary Figure 1: the cell viability of the LO2 cell line treated with HBR. [file 1618491.f1.docx]

**Supplementary Material**


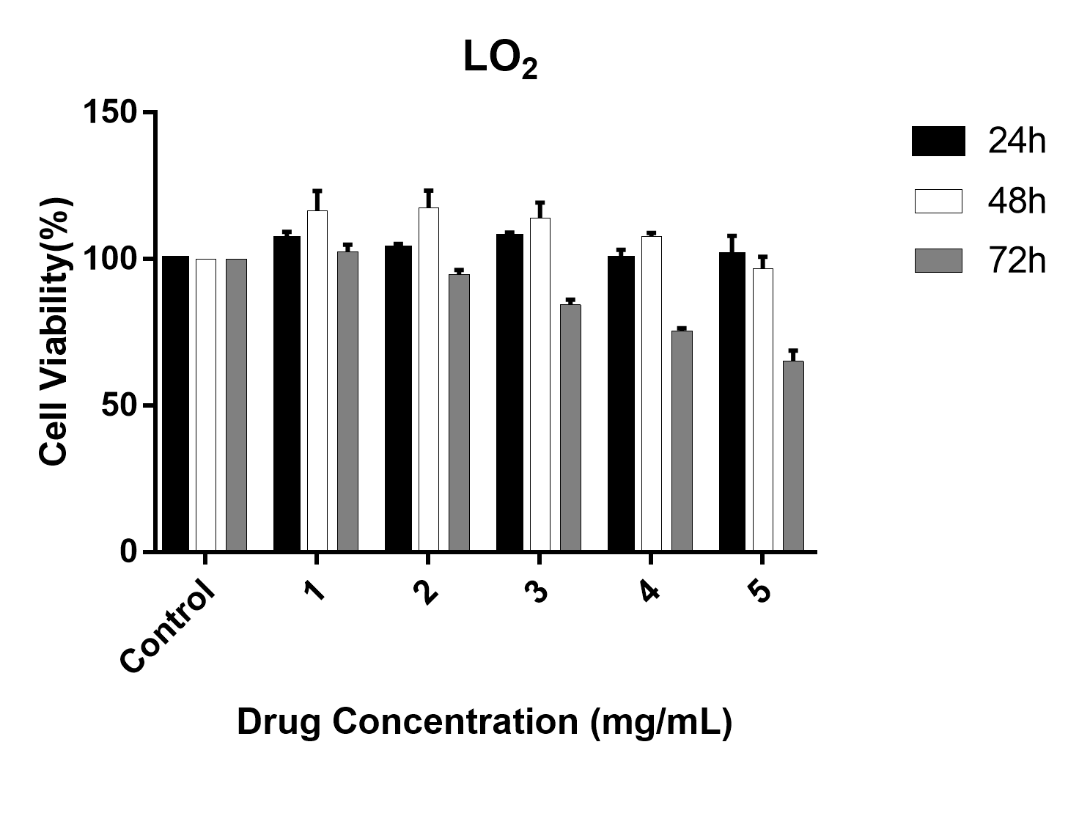


**Supplementary figure 1.** The MTT assay was used to measure the cell viability of the LO_2_ cell line treated with HBR.
